# A Case of Hemolysis, Elevated Liver Enzyme, and Low Platelet Count (HELLP) Syndrome-Precipitated Acute Thrombotic Thrombocytopenia Purpura

**DOI:** 10.7759/cureus.113966

**Published:** 2026-08-04

**Authors:** Aaron Lee, Bryan Pham, Emily Nagler

**Affiliations:** 1 Internal Medicine, San Ysidro Health, San Ysidro, USA; 2 Hematology/Oncology, Scripps Mercy Hospital, San Diego, USA; 3 Hematology and Medical Oncology, Scripps Clinic, San Diego, USA

**Keywords:** congenital ttp, hellp, hellp syndrome, thrombotic thrombocytopenic purpura (ttp), ttp

## Abstract

Hemolysis, elevated liver enzyme, and low platelet count (HELLP) syndrome and thrombotic thrombocytopenic purpura (TTP) are both thrombotic microangiopathies that share overlapping clinical and laboratory features, making differentiation during pregnancy particularly challenging. We present a case of a 27-year-old woman at 21 weeks gestation who initially presented with hypertensive emergency and right middle cerebral artery (MCA) ischemic stroke requiring embolectomy and whose subsequent intensive care unit (ICU) course was complicated by fever, severe thrombocytopenia, microangiopathic hemolytic anemia (MAHA), and transaminitis raising concern for concurrent HELLP syndrome and TTP. Further workup revealed undetectably low ADAMTS-13 activity with negative inhibitor and identification of two heterozygous pathogenic ADAMTS-13 gene variants, confirming a diagnosis of hereditary TTP superimposed on HELLP syndrome. Management was complicated by the need for platelet transfusion prior to delivery despite its contraindication in TTP, which required multidisciplinary collaboration between Ob/Gyn perinatology and hematology. The decision was made to manage concurrent HELLP and TTP with emergent delivery followed by plasma exchange (PLEX).

This unique case demonstrates the challenges of diagnosing and starting appropriate management when there is concern for both HELLP and TTP due to the significant clinical and laboratory overlap. Additionally, a high index of suspicion should be maintained for the possibility of pregnancy precipitating acute-on-chronic hereditary TTP events in patients presenting with severe thrombocytopenia and prior unexplained pregnancy loss.

## Introduction

Hemolysis, elevated liver enzyme, and low platelet count (HELLP) syndrome and thrombotic thrombocytopenic purpura (TTP) are both life-threatening conditions with overlapping clinical and laboratory features. Clinically, both can present with abnormal bleeding, ecchymosis, renal dysfunction, and altered mental status. The two conditions also share key diagnostic findings: hemolysis and thrombocytopenia. Given these similarities, distinguishing between HELLP and TTP is critical, as the emergent management differs substantially. This distinction can be clinically challenging during pregnancy when either condition may occur independently [1]. In rare cases, autoimmune disease, pregnancy, or HELLP can precipitate acute TTP through a mild to moderate decrease in ADAMTS-13 activity [2]. However, in most patients with HELLP syndrome, reduced ADAMTS-13 activity was found to be transient and likely reflects acute liver injury rather than true TTP [3]. We present a case of a pregnant patient with HELLP syndrome and concomitant acute-on-hereditary TTP without prior history of acute TTP.

HELLP is a pregnancy-associated syndrome characterized by hemolysis, elevated liver enzymes, and low platelets. It is driven by immune maladaptation and endothelial damage leading to abnormal vascular function similar to preeclampsia. This placental dysfunction leads to the release of antiangiogenic factors into the maternal circulation, causing endothelial injury, microangiopathy, intravascular coagulation, and subsequent ischemic damage. Patients typically present with hypertension and proteinuria; severe cases can result in disseminated intravascular coagulation or severe hemorrhage from hepatic hematoma rupture. Diagnosis is made by laboratory criteria defined by the American College of Obstetricians and Gynecologists, including lactate dehydrogenase (LDH) > 600 U/L, aspartate aminotransferase (AST) and alanine aminotransferase (ALT) at least twice the upper limit of normal, and platelets < 100,000/µL [4]. Management focuses on stabilization, seizure prophylaxis with magnesium, blood pressure control when indicated, and definitive treatment with prompt delivery.

In contrast, TTP is caused by a deficiency of the von Willebrand factor (vWF)-cleaving enzyme ADAMTS-13. Impaired cleavage of large vWF multimers leads to widespread formation of microvascular thrombi resulting in tissue ischemia throughout the body. TTP is most commonly acquired, approximately 95%, categorized by autoantibody-mediated destruction [5]. Congenital gene mutations coding for ADAMTS-13 lead to hereditary TTP, and although it is a rarer cause of TTP, more than 150 distinct pathogenic mutations have been reported [6]. Clinically, TTP presents with the classic pentad of fever, microangiopathic hemolytic anemia (MAHA), thrombocytopenia, renal dysfunction, and neuropsychiatric symptoms. Diagnosis is confirmed by ADAMTS-13 activity levels, with <10% indicating severe deficiency, but treatment should not be delayed while awaiting results due to the condition’s high mortality if untreated. The PLASMIC (platelet count, hemolysis, active cancer, stem cell or solid organ transplant, mean corpuscular volume, international normalized ratio (INR), and creatinine) score can aid in estimating the likelihood of TTP and guide urgent intervention [7]. Therapeutic plasma exchange (PLEX) is the cornerstone of treatment for TTP, as it replenishes ADAMTS-13 and removes pathogenic autoantibodies. Delivery is currently the standard and only definitive treatment for HELLP; plasma exchange remains controversial and unsupported by adequate studies. Early initiation of PLEX is critical to prevent irreversible neurologic injury, renal failure, cardiac ischemia, and death.

## Case presentation

**Figure 1:**
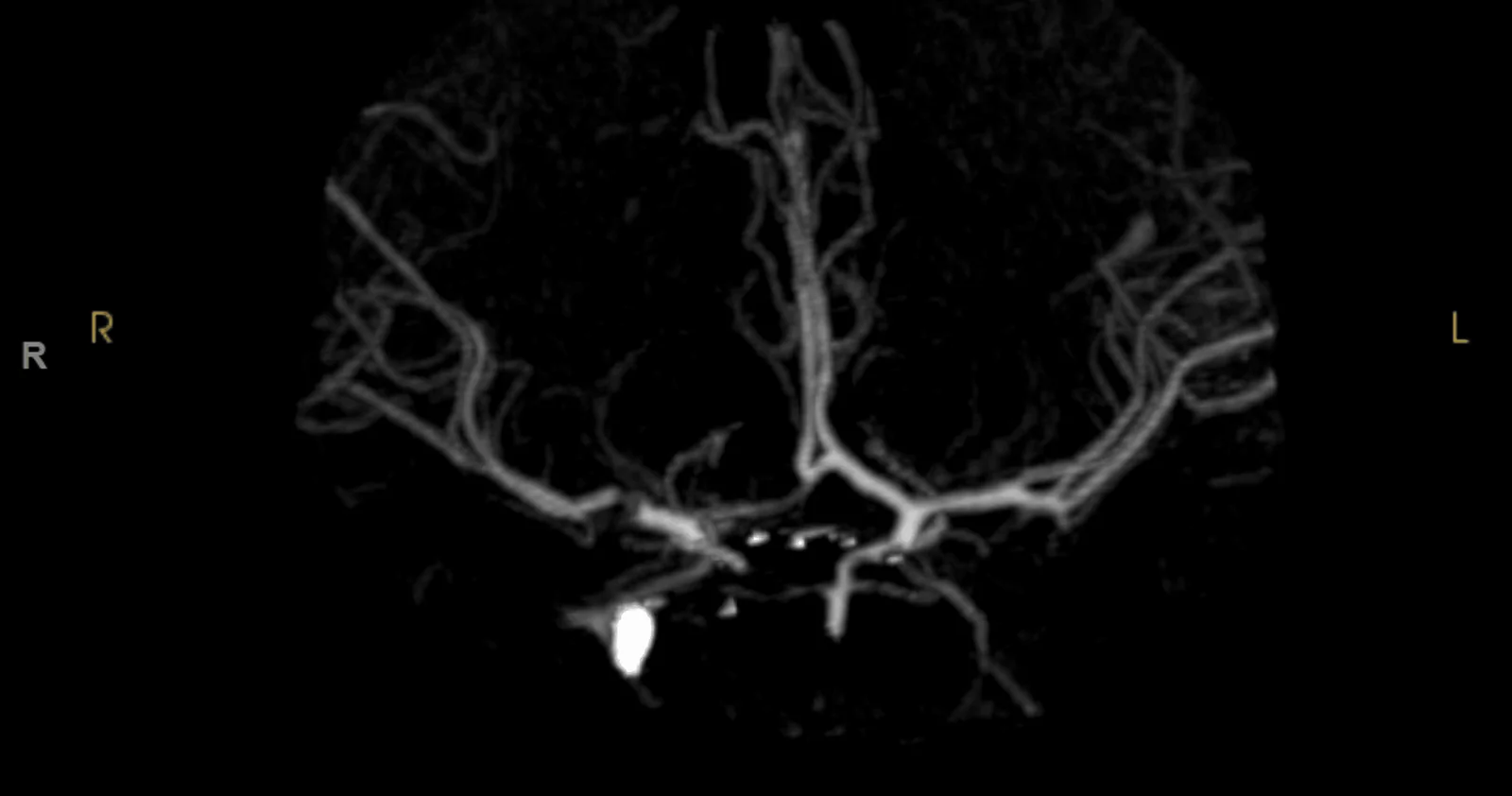
Admission head CT angiography CT angiography demonstrating a right MCA M1 segment occlusion CT: computed tomography, MCA: middle cerebral artery

**Figure 2:**
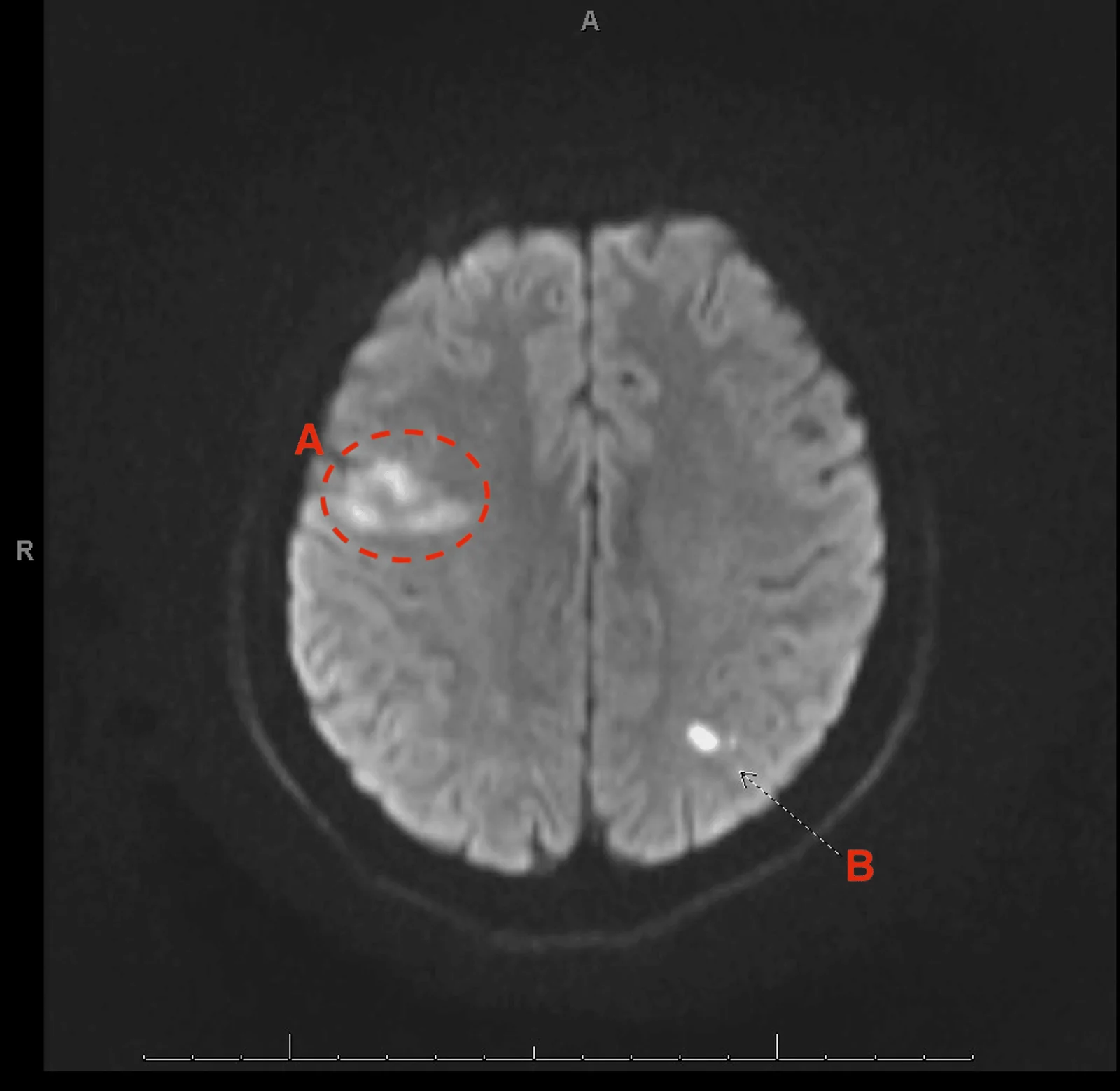
Post-emergent embolectomy MRI of the brain without contrast A: Moderate, subacute, non-hemorrhagic right frontal lobe infarct. Additional small foci of increased T2 FLAIR signal without associated restricted diffusion are identified in the right parietal convexity. These changes are suggestive of old infarcts. Multiplicity and distribution of infarcts are suggestive of an embolic source. B: Small, subacute, non-hemorrhagic left parietal lobe infarcts. MRI: magnetic resonance imaging, FLAIR: fluid-attenuated inversion recovery

Our patient is a 27-year-old G2P0100 female with a history of preeclampsia with previous gestation fetal demise (at 23 weeks gestation) and was 21 weeks gestation at the time, who presented for sudden collapse, left-sided weakness, and slurred speech one hour prior to evaluation in the emergency department. Initial vitals demonstrated an elevated blood pressure of 209/134 and fever with maximum temperature (Tmax) of 100.8°F. Physical examination noted a right gaze deviation and left-sided neglect involving the face. The patient was unable to participate in extremity motor testing. Telemetry showed stable sinus tachycardia. Initial laboratory studies were notable for mild anemia, thrombocytopenia (platelet: 123 K/mcL), transaminitis (aspartate aminotransferase (AST): 68 units/L, alanine aminotransferase (ALT): 47 units/L), and elevated lactate dehydrogenase (LDH) of 1,430 units/L. Computed tomography (CT) of the head and neck angiography demonstrated a right-sided middle cerebral artery (MCA) M1 segment occlusion (Figure 1). Given the patient's intrauterine pregnancy, neurology, neurosurgery, and interventional radiology were consulted and opted for mechanical embolectomy rather than intravenous thrombolytics. A follow-up magnetic resonance imaging (MRI) of the brain after emergent surgery showed moderate subacute right-sided infarcts suggestive of an embolic source and findings suggestive of prior infarcts as well (Figure 2). Although the patient remained neurologically stable without deficits, she was admitted to the intensive care unit (ICU) for close observation.

**Figure 3:**
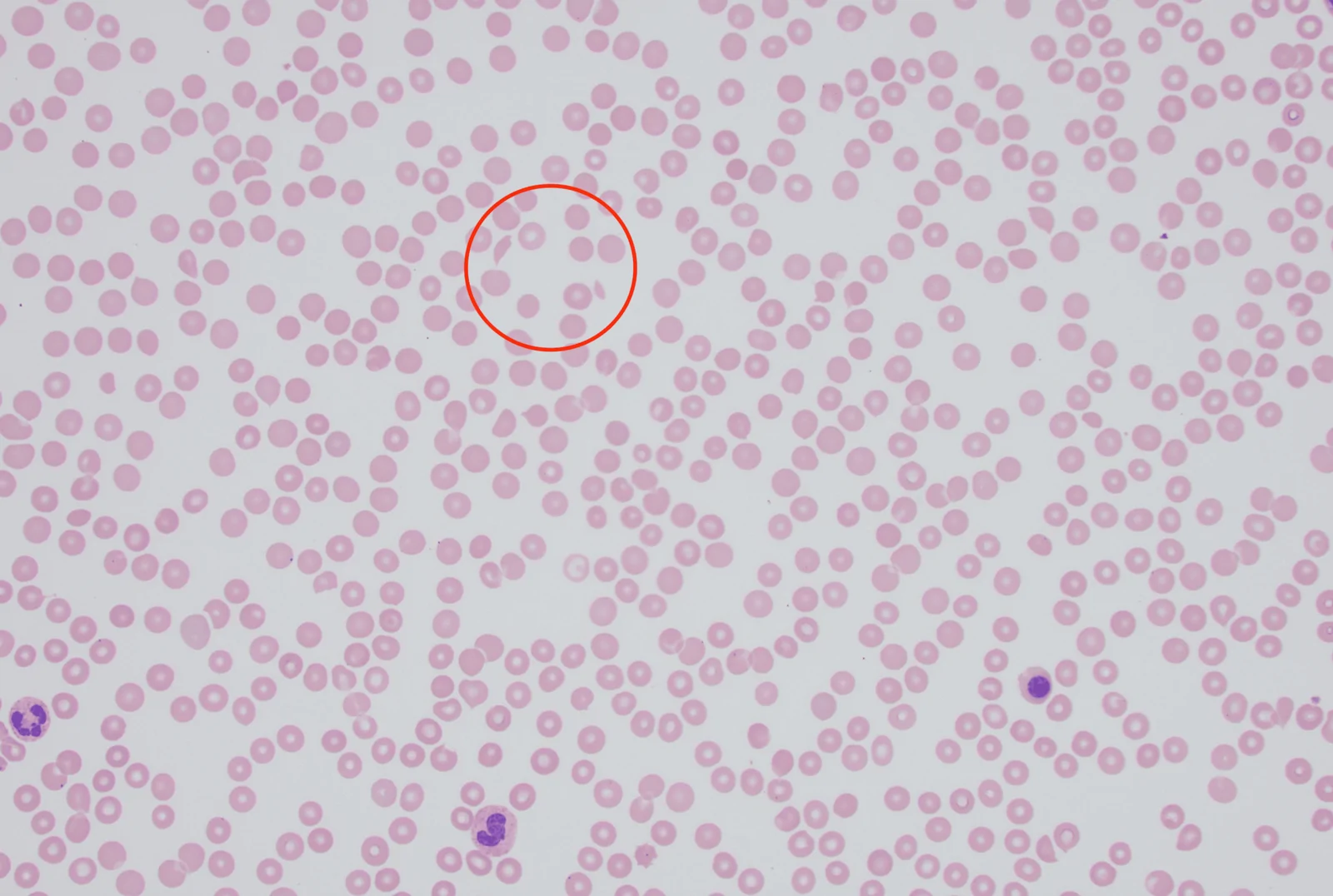
Admission peripheral blood smear The red circle shows rare schistocytes.

Unfortunately, the patient’s ICU course was complicated by sepsis with hemolysis, fever, hematuria, proteinuria, and transaminitis, prompting hematology/oncology consultation for suspected TTP versus HELP syndrome. At the time of consult, her laboratory results showed an increasing LDH of 2,000 units/L, platelets of 14 K/mcL from 123 K/mcL on admission, and worsening transaminitis (AST: 187 units/L, ALT: 110 units/L). Her haptoglobin was <8 mg/dL. A peripheral blood smear showed normocytic anemia with a few schistocytes and severe thrombocytopenia without abnormal platelet morphology or platelet clumping, further suggesting she was developing TTP (Figure 3). Her PLASMIC score was 6, indicating a high risk for TTP and immediate need for PLEX. During this time, hypercoagulable workup including protein C/protein S activity, factor V Leiden mutation, and antiphospholipid testing was without abnormalities with the exception of a slightly low antithrombin activity of 64%. A compilation of the patient’s laboratory workup is presented in Table 1. The patient received multiple blood product transfusions and Ob/Gyn, as well as hematology, recommended plasma exchange, which was arranged by nephrology. She was also started on high-dose methylprednisolone and rituximab. Infectious workup revealed streptococcal pneumonia for which she completed a seven-day course of ceftriaxone. Fortunately, the patient responded to the various therapies employed, resulting in improvement of platelet count without bleeding and resolution of transaminitis. She ultimately received three days of plasma exchange until her platelet count was above 150,000, and she was transitioned to aspirin thereafter. Regrettably, given the continued high suspicion for HELLP, Ob/Gyn recommended emergent induction of labor; surgical pathology confirmed a non-viable fetus and an immature placenta with the fetal surface showing arborizing vasculature, subchorionic fibrin deposition, adherent blood clots, and multiple infarcts. Additionally, due to her severe thrombocytopenia, platelets had to be given prior to labor induction; this further complicated the patient’s clinical course as platelet transfusions are generally contraindicated in TTP due to the risk of thrombotic events [8].

**Table 1:** Laboratory findings on admission compared to time of consultation MCV: mean corpuscular volume, LDH: lactate dehydrogenase, AST: aspartate aminotransferase, ALT: alanine aminotransferase, INR: international normalized ratio

Parameter	Laboratory results on admission	Laboratory results at time of consultation	Reference range
Platelet	123 K/mcL	14 K/mcL	150-425 K/mcL
MCV	92 fL	92 fL	81-100 fL
LDH	1,430 units/L	2,000 units/L	120-246 units/L
AST	68 units/L	187 units/L	14-36 units/L
ALT	47 units/L	110 units/L	≤34 units/L
INR	0.9	1.1	2.0-3.0
Creatinine	0.57 mg/dL	0.54 mg/dL	0.50-1.00 mg/dL
Haptoglobin	-	<8 mg/dL	25-250 mg/dL
Reticulocyte percentage	-	3.51%	0.90%-2.40%
Protein C activity	-	109%	70%-140%
Protein S, functional	-	67%	57%-131%
Antithrombin activity	-	64%	83%-128%
Factor V Leiden mutation	-	Negative	-
Lupus anticoagulant	-	Negative	-
Anti-beta-2 glycoprotein I (IgG, IgA, IgM)	-	<2	<20 antibody not detected
Cardiolipin antibody (IgG, IgA, IgM)	-	<2	<20 antibody not detected

Further testing eventually revealed she had undetectably low ADAMTS-13 levels with a negative ADAMTS-13 inhibitor. A hereditary TTP panel, with a laboratory-stated sensitivity of >99%, showed two heterozygous ADAMTS-13 pathogenic gene variants (c.578G>A and c.768_774dup), further suggesting the patient has congenital TTP in addition to HELLP syndrome. She was discharged on prednisone 60 mg daily indefinitely, started on *Pneumocystis jirovecii* pneumonia (PJP) prophylaxis, and referred to outpatient hematology for additional rituximab infusion.

## Discussion

Pregnant patients presenting with severe microangiopathic hemolytic anemia (MAHA) and thrombocytopenia should be urgently evaluated for emergent causes, including HELLP syndrome, thrombotic thrombocytopenic purpura (TTP), and atypical hemolytic-uremic syndrome (aHUS) [9]. Differentiating between HELLP syndrome and TTP is particularly challenging given their significant clinical and laboratory overlap, as both conditions can present with severe thrombocytopenia, MAHA, renal dysfunction, and neurologic symptoms [10]. A clue that favors TTP over HELLP is the absence of transaminitis, whereas fever, a classic feature of TTP, is not typical of HELLP [11]. The degree of thrombocytopenia can also offer an additional distinguishing feature, as TTP typically presents with more severe thrombocytopenia (platelet count: <30 × 10⁹/L) compared to the generally milder and more variable thrombocytopenia seen in HELLP [6]. ADAMTS-13 activity is a key biomarker in distinguishing the two conditions, as profoundly low levels are the hallmark and pathophysiologic basis of TTP. However, transiently reduced ADAMTS-13 activity can also be seen in isolated HELLP syndrome and is expected to normalize following delivery without requiring plasma exchange (PLEX). Rarely, severe HELLP-induced acute liver failure can impair ADAMTS-13 production enough to precipitate secondary acquired TTP, necessitating PLEX [9]. Thus, while ADAMTS-13 levels support the diagnosis of TTP, an acutely low level alone cannot reliably distinguish TTP from HELLP.

In the unique case of our patient with hereditary TTP, confirmed by persistently undetectable ADAMTS-13 activity and a pathogenic gene variant, she likely presented with concurrent HELLP and acute TTP. Her admission workup showed anemia (hemoglobin: 11 g/dL), thrombocytopenia (platelet: 123 × 10⁹/L), and acute transaminitis, supporting a diagnosis of HELLP. However, during her ICU course, her platelet count drastically declined to 14 × 10⁹/L and was found to have an undetectably low haptoglobin, which raised concern for acute TTP as well. Prompt interdisciplinary discussion between Ob/Gyn perinatology, hematology, and the ICU was initiated to determine whether the patient had isolated TTP, isolated HELLP, or a concurrent episode of both conditions. This distinction made appropriate treatment particularly challenging.

While prompt delivery is the definitive treatment for HELLP, this patient was also severely thrombocytopenic and would require platelet transfusion prior to delivery. Platelet transfusions are not routinely recommended in TTP, as they are associated with higher odds of arterial thrombosis and mortality [8]. Given the high risk for decompensation, the decision was made to proceed with platelet transfusion to a safe threshold while monitoring closely for neurologic or EKG changes suggestive of thromboembolic events prior to urgent delivery. PLEX was initiated as soon as possible following delivery. Standard PLEX consists of a minimum of 2,000 mL per session (40-60 mL/kg) administered daily or twice daily [12]; this patient received daily PLEX with 3,250 mL of fresh frozen plasma for three days. She additionally received high-dose methylprednisolone and completed four doses of rituximab. Upon completion of therapy, her platelet count improved to 164 × 10⁹/L with no significant change in hemoglobin, and her acute liver injury resolved.

Pregnancy is a well-recognized trigger for acute episodes of hereditary TTP, as it further reduces physiologic ADAMTS-13 activity while simultaneously increasing von Willebrand factor (vWF) concentration, creating a prothrombotic state that exacerbates the already severely deficient ADAMTS-13 activity in affected patients [13]. ADAMTS-13 levels may decline by 70%-75% from baseline by the end of pregnancy, and the placenta itself appears particularly vulnerable to ADAMTS-13 deficiency, with maternal vascular infarcts commonly observed [5]. Without prophylactic treatment, patients with hereditary TTP face an approximately 100% risk of an acute episode during subsequent pregnancies [14]. The risk during a first pregnancy, while substantial, is less precisely quantified in current literature. Early recognition is critical, as PLEX initiated at the time of confirmed pregnancy can prevent acute TTP attacks and fetal complications such as growth restriction [14]. Our patient was not diagnosed with TTP during her first pregnancy, which resulted in fetal loss attributed to presumed preeclampsia at the time. However, with further workup, she most likely had an unrecognized initial episode of acute-on-chronic hereditary TTP, and her second pregnancy led to full manifestation of the disease.

Patients with hereditary TTP require ongoing monitoring, including ADAMTS-13 activity, approximately every three months to guide management and prevent future acute episodes [15]. Following discharge, the patient continued to follow up with Hematology every three months with repeated PLEX and was found to have recovered her ADAMTS-13 activity to 23%. She fortunately did not suffer any lasting neurologic sequelae from her right MCA ischemic stroke. Hematology also referred her to genetic counseling for the ADAMTS-13 pathogenic gene variants and advised her to consider surrogacy for future family planning.

## Conclusions

This case highlights the diagnostic and therapeutic challenges of managing thrombotic microangiopathies during pregnancy, particularly when HELLP syndrome and hereditary TTP present concomitantly due to their multitude of overlapping clinical and laboratory features. Pregnancy can precipitate acute TTP episodes in patients with underlying ADAMTS-13 deficiency, and reliance on ADAMTS-13 activity alone may be insufficient to distinguish isolated HELLP from concurrent TTP in an emergent setting. Early multidisciplinary collaboration and prompt initiation of plasma exchange are critical to prevent maternal morbidity and adverse fetal outcomes. This case underscores the importance of maintaining a high index of suspicion for hereditary TTP in pregnant patients with severe thrombocytopenia and microangiopathic hemolytic anemia, especially in those with prior pregnancy complications, as prompt recognition and timely intervention can be lifesaving and inform long-term management and family planning decisions.
